# Effects of the CrossFit Exercise Data Analysis on Body Composition and Blood Profiles

**Published:** 2017-09

**Authors:** Eun-Ju CHOI, Wi-Young SO, Taikyeong Ted. JEONG

**Affiliations:** 1. Division of Sport Sciences, Konkuk University, Chungju-si, Korea; 2. Sports and Health Care Major, Korea National University of Transportation, Chungju-si, Korea; 3. Dept. of Computer Science and Engineering, Seoul Women’s University, Seoul, Korea

## Dear Editor-in-Chief

Most of college students, despite their commonly perceived good health, report experiencing medical conditions, such as attention-deficit/hyperactivity disorder, learning disabilities, psychiatric disorders, and other chronic illnesses (1–2).

Previous research has shown that exercise training results in consistent improvements in health status, including that of mental health (3–4). Recently, CrossFit exercise has been able to improve dramatically the aerobic capacity, anaerobic power, health-related fitness, body composition, and presence of neurotrophic factors in users of the program (5–7). However, few, if any, scientific studies have investigated the effectiveness of CrossFit exercise in college students.

Therefore, the purpose of this study was to examine the effects of the CrossFit exercise program on body composition and blood profiles in Korean college students.

The present study included 22 college students who visited the Exercise Physiology Laboratory in Korea National University of Transportation, to undergo measurements of body composition and blood profiles. The participants were divided randomly into the CrossFit exercise group (male=6, female=5; age, 19.82±1.72 yr; height, 168.79±7.65 cm; weight, 65.13±11.54 kg) or the control group (male=6, female=5; age, 19.09±0.94 years; height, 167.95±9.15 cm; weight, 62.16±8.73 kg). Before participating in this study, all participants provided written informed consent.

Participants underwent body composition and blood profile assessment before and after intervention. The CrossFit group performed a Cross-Fit program for 70 minutes, two times per week for 14 weeks, which is a modified version of the exercise protocol (7). Meanwhile, the control group was asked not to perform special or regular physical activity, except for activities of daily living for 14 weeks.

The parameters of body composition (i.e., weight, body mass index, muscle mass, percent body fat, and basal metabolic rate) were measured using Inbody-720 equipment (Biospace, Seoul, Korea). For each subject, 2–3mL of blood was obtained from a forearm vein in the morning after a 12-h fast using a vacuum blood-gathering tube. The blood profiles(i.e., aspartate aminotransferase, alanine aminotransferase, gamma-glutamyl transferase, total bilirubin, total cholesterol, low density lipoprotein cholesterol, high density lipoprotein cholesterol, triglycerides, glucose, total protein, albumin, blood urea nitrogen, and creatinine levels) were measured using IVDA10A automated analyzer(P/N:F00Q4-0043K-01; S/N: C004M1ABB00032X, Samsung, Korea).

All data are presented as the mean ± standard deviation. Data analysis was performed using a 2 × 2(group × time) repeated measures analysis of variance. All analyses were performed using SPSS Ver. 18.0(SPSS, Chicago, IL, USA). Statistical significance was assumed when *P*<0.05.

Interaction effects (time x group) on weight (*P*=0.003), body mass index (*P*=0.002), muscle mass (*P*=0.007), and basal metabolic rate (*P*=0.010) were observed. However, there were no interaction effects (time x group) for any values obtained in blood profiles (*P*>0.05) (Table 1).

**Table 1:** Changes in body composition and blood profiles after 14 weeks of the CrossFit exercise program

**Variables**	**Group**	**Pre**	**Post**	**Interaction (group × time)**	
				**F**	***P***
**Weight (kg)**	Cross-fit	65.13±11.54	64.15±11.76	11.327	0.003**
	Control	62.16±8.73	62.82±8.54		
**Body mass index (kg/m^2^)**	Cross-fit	22.70±2.58	22.35±2.65	12.315	0.002**
	Control	21.98±2.11	22.23±2.26		
**Muscle mass (kg)**	Cross-fit	28.42±7.30	28.48±7.24	8.956	0.007**
	Control	27.29±6.69	28.01±6.68		
**Body fat (%)**	Cross-fit	22.51±6.78	21.20±6.40	0.058	0.813
	Control	21.79±9.09	20.67±9.25		
**Basal metabolic rate (kcal)**	Cross-fit	1466.73±251.69	1468.27±252.41	7.997	0.010*
	Control	1426.73±231.25	1452.18±227.43		
**Aspartate aminotransferase (U/L)**	Cross-fit	23.73±6.18	23.91±9.51	0.421	0.524
	Control	19.82±9.55	18.45±5.82		
**Alanine aminotransferase (U/L)**	Cross-fit	27.91±13.26	24.64±10.24	1.443	0.244
	Control	20.18±7.14	19.64±7.37		
**Gamma-glutamyl transferase (U/L)**	Cross-fit	25.55±6.77	24.73±5.39	0.010	0.923
	Control	23.64±3.17	23.00±3.85		
**Total bilirubin (mg/dl)**	Cross-fit	0.54±0.14	0.55±0.34	2.478	0.131
	Control	0.97±0.46	0.74±0.36		
**Total cholesterol (mg/dl)**	Cross-fit	136.18±30.90	125.82±39.00	1.058	0.316
	Control	148.18±34.64	118.27±40.88		
**Low density lipoprotein cholesterol (mg/dl)**	Cross-fit	72.73±22.19	71.91±25.28	1.155	0.295
	Control	79.09±25.96	66.45±23.13		
**High density lipoprotein cholesterol (mg/dl)**	Cross-fit	50.18±12.06	44.09±16.82	1.281	0.271
	Control	56.82±12.82	42.09±16.51		
**Triglycerides (mg/dl)**	Cross-fit	66.45±30.60	48.82±22.47	0.066	0.800
	Control	62.00±21.94	47.73±21.67		
**Glucose (mg/dl)**	Cross-fit	87.73±10.75	81.91±20.61	0.276	0.605
	Control	89.64±11.39	78.27±24.69		
**Total protein (g/dl)**	Cross-fit	7.35±0.60	6.35±1.24	0.093	0.764
	Control	7.29±0.57	6.09±1.23		
**Albumin (g/dl)**	Cross-fit	4.97±0.47	4.63±0.87	0.675	0.421
	Control	5.10±0.54	4.36±1.04		
**Blood urea nitrogen (mg/dl)**	Cross-fit	11.12±3.86	10.30±3.82	0.378	0.546
	Control	9.41±3.44	10.18±6.66		
**Creatinine (mg/dl)**	Cross-fit	0.78±0.16	0.82±0.18	0.008	0.929
	Control	0.82±0.11	0.87±0.19		

Data are presented as means±standard deviations

Cross-fit group, n=11; control group, n=11

* *P*<0.05,

** *P*<0.01; tested by repeated measure analysis of variance

We concluded that 14 weeks of supervised CrossFit exercise is effective in modifying body composition; however, it is not effective in modifying blood profiles in a sample of Korean college students. In future, well-designed studies, which include a larger sample size and the inclusion of additional exercise groups, are necessary.
